# Small Intestine-specific Knockout of CIDEC Improves Obesity and Hepatic Steatosis by Inhibiting Synthesis of Phosphatidic Acid

**DOI:** 10.7150/ijbs.74348

**Published:** 2022-09-11

**Authors:** Liang Huang, Qichao Liao, Tingli Pan, Yu Sun, Zhier Aluo, Lianggui Xiao, Jingsu Yu, Siqi Liu, Yang Xiao, Yufeng Yang, Yixing Li, Lei Zhou

**Affiliations:** State Key Laboratory for Conservation and Utilization of Subtropical Agro-bioresources, College of Animal Science and Technology, Guangxi University, Nanning, China.

**Keywords:** obesity, hepatic steatosis, small intestine, CIDEC, lipid absorption phosphatidic acid

## Abstract

The small intestine is main site of exogenous lipid digestion and absorption, and it is important for lipid metabolic homeostasis. Cell death-inducing DNA fragmentation-factor like effector C (CIDEC) is active in lipid metabolism in tissues other than those in the intestine. We developed small intestine-specific CIDEC (SI-CIDEC^-/-^) knockout C57BL/6J mice by Cre/LoxP recombination to investigate the *in vivo* effects of intestinal CIDEC on lipid metabolism. Eight-week-old SI-CIDEC^-/-^ mice fed a high-fat diet for 14 weeks had 15% lower body weight, 30% less body fat mass, and 79% lower liver triglycerides (TG) than wild-type (WT) mice. In addition, hepatic steatosis and fatty liver inflammation were less severe in knockout mice fed a high-fat diet (HFD) compared with wild-type mice fed an HFD. SI-CIDEC^-/-^ mice fed an HFD diet had lower serum TG and higher fecal TG and intestinal lipase activity than wild-type mice. Mechanistic studies showed that CIDEC accelerated phosphatidic acid synthesis by interacting with 1-acylglycerol-3-phosphate-O-acyltransferase to promote TG accumulation. This study identified a new interacting protein and previously unreported CIDEC mechanisms that revealed its activity in lipid metabolism of the small intestine.

## Introduction

Obesity is a global epidemic disease that seriously endangers human health. In 2016, the World Health Organization estimated that the prevalence of obesity, a body mass index ≥30 kg/m^2^, was 36.2% in the United States, 29.4% in Canada, 29% in Australia, 28.9% in Mexico, and 23.1% in Russia 1. Obesity is caused by an imbalance of fat metabolism that involves the intestine. The apical membrane of the intestinal villi absorbs digested lipids and circulating lipids are transported from the blood circulation through the basal membrane for catabolism in the intestinal cells 2-4. Lipases break down exogenous lipids in the intestinal lumen into small molecules such as 2-monoacylglycerols (MAGs), fatty acids (FAs), lipoprotein lipases (LPLs) and cholesterol esters (CEs). FAs can be actively transported in the presence of proteins such as CD36, scavenger receptor class B type 1 (SR-B1), and fatty acid transport protein (FATP) 4, but mainly rely on FAs, MGs, and LPL micelles with bile acid salts to enter the intestinal epithelium 5-8. After entering the cell, lipid products of digestion are transported into the endoplasmic reticulum (ER) where they are re-esterified by alpha-1,3-mannosyl-glycoprotein 2-beta-N-monoacetylglyceride acyltransferases (MGATs) and diglyceride acyltransferases (DGATs) to form triacylglycerols (TGs) that are used to form lipid droplets for storage in the cytoplasm or for chylomicron synthesis. Mature chylomicrons are transported by lymph into the blood and are a source of lipids for peripheral tissues, especially the liver.

CIDEC (cell death-inducing DNA fragmentation-factor like effector C), also known as fat specific protein (Fsp)27, was initially found to be associated with the adipose differentiation of 3T3-L1 cells 9. The CIDEC protein promotes formation of unilocular lipid droplets 10-12. In 2015, an Fsp27 isoform, Fsp27β/CIDEC-l, which has 10 more amino acids than Fsp27α/CIDEC-s was described 13. Alternative start codons are responsible for the formation of different splices of CIDEC 13. CIDEC-l is highly expressed in steatotic livers. The functions of CIDEC in the liver, adipose tissue, and the whole body have been described 11-17, but the role of CIDEC in the lipid metabolism of the small intestine is not clear. We previously reported that the longer isoform is the one that is primarily expressed in the small intestine in pigs 18. We investigated CIDEC activity in the homeostasis of lipid metabolism in small intestine-specific CIDEC knockout (SI-CIDEC^-/-^) mice. After 14 weeks of a high-fat diet (HFD), the knockout mice weighed less and had less fat than wild-type (WT) mice. Loss of CIDEC function alleviated diet-induced obesity and hepatic steatosis. The study revealed that intestinal CIDEC regulated lipid metabolism homeostasis, and provided a rationale for subsequent research.

## Material and Methods

### Animals

Small intestine-specific knockout CIDEC mice (SI-CIDEC^-/-^) were obtained by backcrossing CIDEC^flox/flox^ mice in which a loxP site is inserted at both ends of exon 3 of CIDEC, with Villus-Cre mice heterozygous for C57BL/6J as a background. SI-CIDEC^-/-^ mice were Villus-Cre mice with homozygous CIDEC^flox/flox^. Homozygous CIDEC^flox/flox^ mice without Villus-Cre were used as WT control mice. Real-time polymerase chain reaction (RT-PCR) was performed with the primers 5′-AAATACTCTATGGCTGCCTCCCC-3′, 5′-TGTCTAACAAGTCCCCAAACTGT-3′ to separate CIDEC^flox/flox^ (200 bp) and the WT gene (139 bp) in mouse offspring with Villus-Cre. Both CIDEC^flox/flox^ and Villus-Cre mice were purchased from Cyagen (Suzhou, China). The mice were male and were housed at 22-24°C in a 12 h light/dark cycle. Mice from the same litter were randomly assigned to groups of 8-10 animals each. There were no significant differences in the mean body weights between the groups. To model obesity, mice were fed an HFD, 60% kcal from fat, produced by Trophic Animal Feed High-Tech Co; Haian, China). A normal diet (ND) was the control. Body composition was recorded once weekly for 9 weeks after modeling. Mice were fasted for 12 h at 22 weeks of age (i.e., 14 weeks of modeling) and then killed by breaking their necks. Eye-vein blood, liver tissue, epididymal fat, and subcutaneous fat were collected. The animal procedures were performed following the guidelines, and with approval, of the animal experimental ethics guidelines of Guangxi University (no. GXU-2021-120).

### Computed tomography (CT)

22-week-old mice were fasted for 6 h, anesthetized by injection of 1.25% tribromoethanol at 10 μl/g body weight, and evaluated by an X-ray Safety Report Skyscan 1278 (Bruker; Rheinstetten, Germany) for adipose tissue distribution, which was an alyzed by the company's supporting software to label the fat as red 19.

### Energy expenditure

Energy metabolism, CO_2_ production, oxygen consumption, and activity were measured every 5 min for 2 days with a Promethion ∞RE Metabolic Analysis System CGF (Sable Systems International; North Las Vegas, NV, USA) as previously descibed 20.

### Glucose tolerance (GTT) and insulin tolerance (ITT) tests

GTTs were performed in 15-week-old mice after a 16 h fast. Mice fed an HFD or an ND were injected with a 20% glucose solution at 5 μL/g body weight, and the blood glucose concentration was measured and recorded at 0, 15, 30, 60, 90, and 120 min. ITTs were performed in 18-week-old mice after fasting for 6 h. Mice fed an HFD or ND were injected with insulin 0.5 U/kg body weight, and the blood glucose concentration was determined as for the GTT.

### Histology

Tissue was stored in 4% paraformaldehyde for 24 h, passed through a 12%, 20%, and 30% sucrose series for 12 h per step, and embedded in Tissue-Tek OCT compound (Sakura; Oakland, CA, USA), and 5 µm frozen sections were cut at -25°C with a microtome (Leica Biosystems, Shanghai, China). The sections were mounted on glass slides, stained with hematoxylin and eosin or oil red O following the manufacturer's (Beyotime; Shanghai, China) protocol. The sections were cover slipped with resin, and photographed with a Nikon 90i microscope using a 40× objective.

### Postprandial TG secretion

SI-CIDEC^-/-^ and WT mice fed an HFD or ND were fasted overnight (16 h), and 10 min after injecting tyloxapol (MedChemE-xpress; Shanghai, China) 500 mg/kg, olive oil gavage were carried out at 15 uL per gram of body weight, blood was collected from the tail vein of 21-week-old mice at 0-6 h post-gavage and used to measure serum TG.

### Serum and tissue biochemical analysis

#### Sample preparation

Small intestinal tissue was divided into three equal segments of the proximal (duodenum), middle (jejunum), and distal (ileum) sections. Two centimeters of each section were removed, flushed with prechilled phosphate buffered saline and placed in RIPA buffer (Solarbio; Beijing, China). After lysis, the samples were centrifuged at 4 °C for 10 min at 12 000 rpm, and the supernatant was taken for assaying. Liver tissue samples were prepared in the same way. Blood samples were rested at 4 °C for 1 h and centrifuged at 3000 rpm for 20 min at 4 °C, and the serum supernatant was collected.

#### Biochemical assays

TGs, total cholesterol (TC), serum lipase (LPS), alanine transaminase (ALT), aspartate aminotransferase (AST), high-density lipoprotein cholesterol (HDL-C) and low-density lipoprotein cholesterol (LDL-C) were assayed with commercially available kits (Njjcbio; Nanjing, China) following the manufacturer's instructions. Malonaldehyde (MDA) was assayed with a lipid peroxidation kit and protein concentration was assayed with a bicinchoninic acid assay kit (both from Beyotime; Shanghai, China). Intestinal tissue phosphatidic acid was assayed with a mouse ELISA kit (Coiba Bio; Shanghai, China). Optical density was measured with an Infinite M200 Pro microplate reader (Teacan; Männedorf, Switzerland). All values were normalized to protein concentrations.

### IPEC-J2 intestinal porcine epithelial cells

IPEC-J2 cell were cultured in complete Dulbecco's minimal Eagle medium (DMEM containing 1% cyanine-streptomycin and 10% fetal bovine serum and high-fat induction by DMEM containing 0.4% oleic acid, 0.2% palmitic acid, 1% cyanine-streptomycin, 2% bovine serum albumin at 37 °C and 5% CO_2_. Cells were transfected with pcDNA3.1(-)-CIDEC-l (sus) plasmids or empty vectors were mixed with liposome following the manufacturer's protocol (Yesen; Shanghai, China). The liposomes complexes were transfected into IPEC-J2 cells cultured in serum-free DMEM. After 4-6 h, the medium was changed to OAPA. Cell samples were collected after 48 h. Cell lysates were prepared using RIPA buffer as described for tissues.

### RNA isolation and RT-PCR

RNA was isolated with Trizol reagent (Invitrogen; Solarbio; Beijing, China). cDNA was reverse transcribed from 1 µg RNA by M-MLV reverse transcriptase (Promega, Beijing, China). RT-PCR assays were performed using Real Star Green Fast mixture with ROXII (GeneStar; Beijing, China) and a qTOWER3G thermal cycler (Analytik; Jena, Germany). The primer sequences are listed in Table S1. For statistical analysis of mRNA expression, values were calculated using the 2^-ΔΔCT^ method.

### 16S rRNA sequencing of gut microbes

Fresh feces obtained from six 22-week-old mice fed an HFD were used. The full length 16S gene (27bp-1492bp) was amplified using the primers: 5′-AGAGTTTGATCCTGGCTCAG-3′; 5′-AGAGTTTGATCCTGGCTCAG-3′. Sequencing and statistical analysis were performed by Biozeron (Shanghai, China).

### Western blotting

Protein supernatant concentration was measured by the bicinchoninic acid method. Samples were added to protein loading buffer and boiled for 10 min at 100 °C. Anti-CIDEC (1:2000; Cloud-Clone Corp; Wuhan, China) and anti-alpha tubulin (Beyotime; Shanghai, China) were the primary antibodies. The blots were developed using HRP-conjugated secondary antibodies (1:3000) and an enhanced chemoluminescence-plus system (Bio-rad; California, USA).

### Co-immunoprecipitation and mass spectrum (Co-IP/MS)

Small intestine tissue was collected from WT mice (no flox). Intestinal protein supernatants were prepared and the protein concentration was assayed as described above. The supernatants were immunoprecipitated with a polyclonal CIDEC antibody (Cloud-Clone Corp; Wuhan, China) following the manufacturer's protocol. Briefly, 3 mg protein supernatant samples were precleared by IgG and arotein L-agarose (Santa Cruz Biotechnology; Dallas, TX, USA) for 30 min, incubated with 2 mg of primary antibody for 1h and then incubated with protein L-agarose at 4 °C overnight (16 h). The bound proteins were washed twice with prechilled RIPA and eluted by boiling in Sodium dodecyl-sulfate polyacrylamide gel electrophoresis (SDS-PAGE) loading buffer (Solarbio; Beijing, China). The resulting proteins were assayed by SDS-PAGE and sent to APTBIO (Shanghai, China) for mass spectrum analysis.

### Kyoto Encyclopedia of Genes and Genomes (KEGG) and genome database enrichment analysis

The proteins identified by mass spectrum were included in a FASTA database that was used for KEGG pathway enrichment analysis, which was performed by the online biological tool KOBAS 3.0.

### Statistical analysis

The results were reported as means ± standard error of the mean (SEM) analyzed by Excel using unpaired *t*-tests or One-way analysis to calculate significance. *P-*values < 0.05, < 0.01, and < 0.001 were considered significant.

## Results

### SI-CIDEC^-/-^ mice were resistant to diet-induced obesity

**Figure 1:**
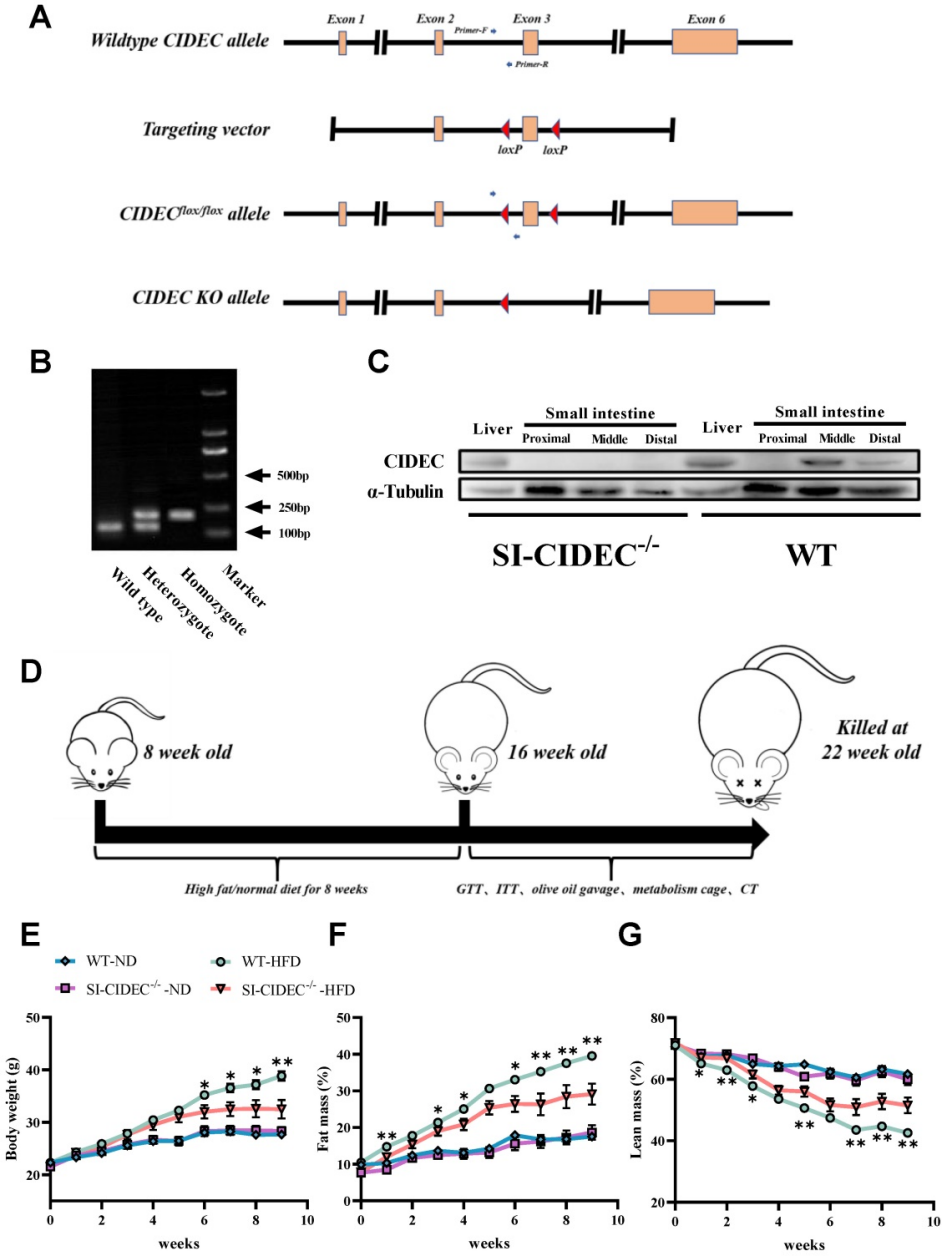
** Generation and modeling of small intestine-specific knockout CIDEC mice. (A)** Genotyping strategy of CIDEC^flox/flox^ and SI-CIDEC^-/-^ mice. **(B)** Genotype identification by AGE (agarose gel electrophoresis) for distinguishing mice with homozygous CIDEC^flox/flox^ from wild-type CIDEC (no flox) and heterozygous CIDEC^flox/flox^; sites of primers are shown in Fig 1A; **(C)** Western blots of CIDEC protein of the liver and small intestine in SI-CIDEC^-/-^ WT mice;** (D)** Workflow of establishing high-fat model mice. **(E-G)** Body composition of mice during 0-9 weeks after modeling, mice modeling from 8 weeks of age; WT mice fed a normal diet (WT-ND, n=10), SI-CIDEC^-/-^ mice fed an ND (SI-CIDEC^-/-^-ND, n=7), WT mice fed a high-fat diet (WT-HFD, n=7) and SI-CIDEC^-/-^ mice fed a high-fat diet (SI-CIDEC^-/-^-HFD, n=7) were used. Data are means ± SEM. Differences of WT versus SI-CIDEC^-/-^ mice fed an HFD were considered significant at *p< 0.05, **p< 0.01.

**Figure 2:**
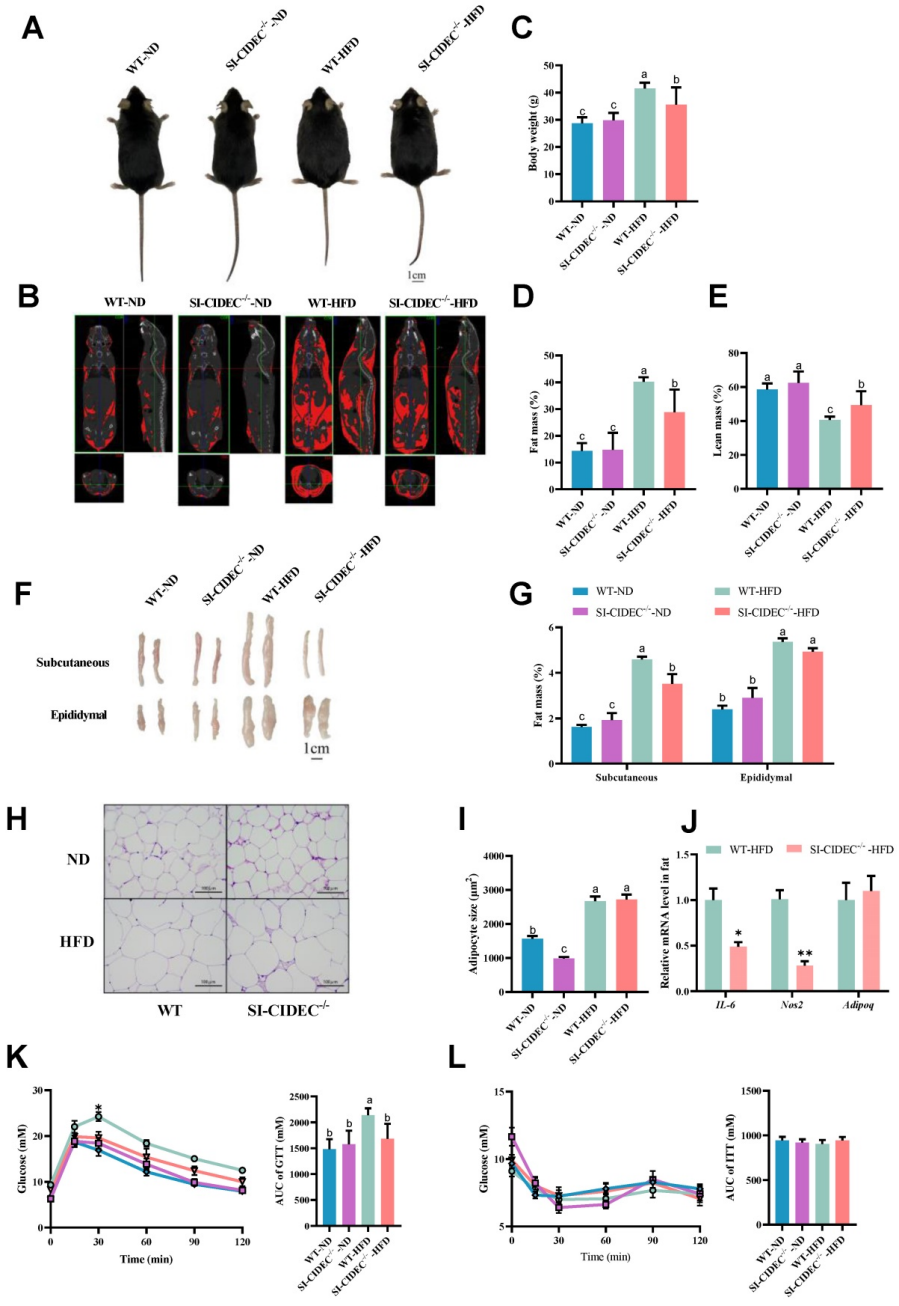
** SI-CIDEC^-/-^ mice were resistant to diet-induced obesity. (A-K) 22-week-old mice fed high-fat or normal diets mice were modeled beginning at 8 weeks of age used. (A)** Representative photographs of mice; Scale bar: 1cm;** (B)** Representative computed tomography (CT) scan analysis. Fat is shown in red; **(C-E)** Body composition (n=7-10); **(F)** Representative photographs of adipose tissue; Scale bar: 1 cm; **(G)** Weight of subcutaneous and epididymal adipose tissue (n=7-10); **(H)** Representative photographs of adipose tissue histology and **(I)** adipocyte area; stained by hematoxylin and eosin. Scale bar: 100 µm (40× objective); **(J)** mRNA of inflammation-related genes, n=3-4; **(K)** Serum glucose concentrations of 15-week-old mice in the glucose tolerance tests (GTTs) (n=6-10); Differences were considered significant at *p < 0.05.** (L)** Serum glucose concentrations of 18-week-old mice in insulin tolerance tests (ITTs, n=6-10). Data are means ± SEM. Mean values with different superscript letters differ significantly (*p* < 0.05).

To obtain SI-CIDEC^-**/**-^ mice, loxP sites were inserted at both ends of exon 3 of CIDEC in CIDEC^flox/flox^ mice (Figure 1A), primers on both ends of the first loxP site were used for PCR, and agarose gel electrophoresis was used to distinguish heterozygous from homozygous CIDEC^flox/flox^ mice (Figure 1B). PCR identified CIDEC with a loxP site of 200 bp, while that of the WT CIDEC was 139 bp. CIDEC^flox/flox^ mice were crossed with Villus-Cre mice to generate small intestine-specific (SI-CIDEC^-**/**-^) mice. In SI-CIDEC^-**/**-^ mice, exon 3 of CIDEC, consisting of 154 bp, was knocked out, which caused a code-shift mutation and both two isoforms of CIDEC were knockout. As a result, CIDEC protein was not detected in SI-CIDEC^-**/**-^ mouse intestine, but no change was seen in the liver (Figure 1C). To model obesity, SI-CIDEC^-**/**-^ and WT mice were fed an HFD and controls were fed an ND (Figure 1D). As shown in (Figure 1E-G), SI-CIDEC^-**/**-^ mice had significantly lower mean body weight and fat mass than WT mice on the HFD at 6 weeks post-modeling. At 14 weeks after HFD induction, the SI-CIDEC^-**/**-^ mice had reduced body size and fat mass (Figure 2A, B). Body composition analysis showed that SI-CIDEC^-**/**-^ -HFD mice had 15% lower body weight and 30% lower fat mass compared to WT-HFD mice (Figure 2C-E). Consistent with those results, the weights of subcutaneous and epididymis adipose tissue were reduced by 23.4% and 25.1% respectively (Figure 2F, G). No difference was found in adipocyte size in SI-CIDEC^-**/**-^-HFD and WT-HFD mice (Figure 2H, I). Interestingly, SI-CIDEC^-**/**-^-ND and WT mice had similar fat masses, but adipocyte size was reduced by 37.07% in the SI-CIDEC^-**/**-^-ND mice compared with the latter. Subsequently, we measured the mRNA expression of inflammation-related genes in adipose tissue. The results showed that the mRNA expression of both IL-6 and Nos2 was significantly decreased (Figure 2J). As in CIDEC-deficient mice 11, glucose tolerance testing found that SI-CIDEC^-**/**-^-HFD mice had increased glucose tolerance compared with those fed an ND (Figure 2K). Differences in the insulin tolerance test results obtained in the SI-CIDEC^-**/**-^ and WT mice fed either the HFD or ND were not significant (Figure 2L).

### SI-CIDEC^-/-^ improved diet-induced hepatic steatosis and hyperlipidemia risk

**Figure 3:**
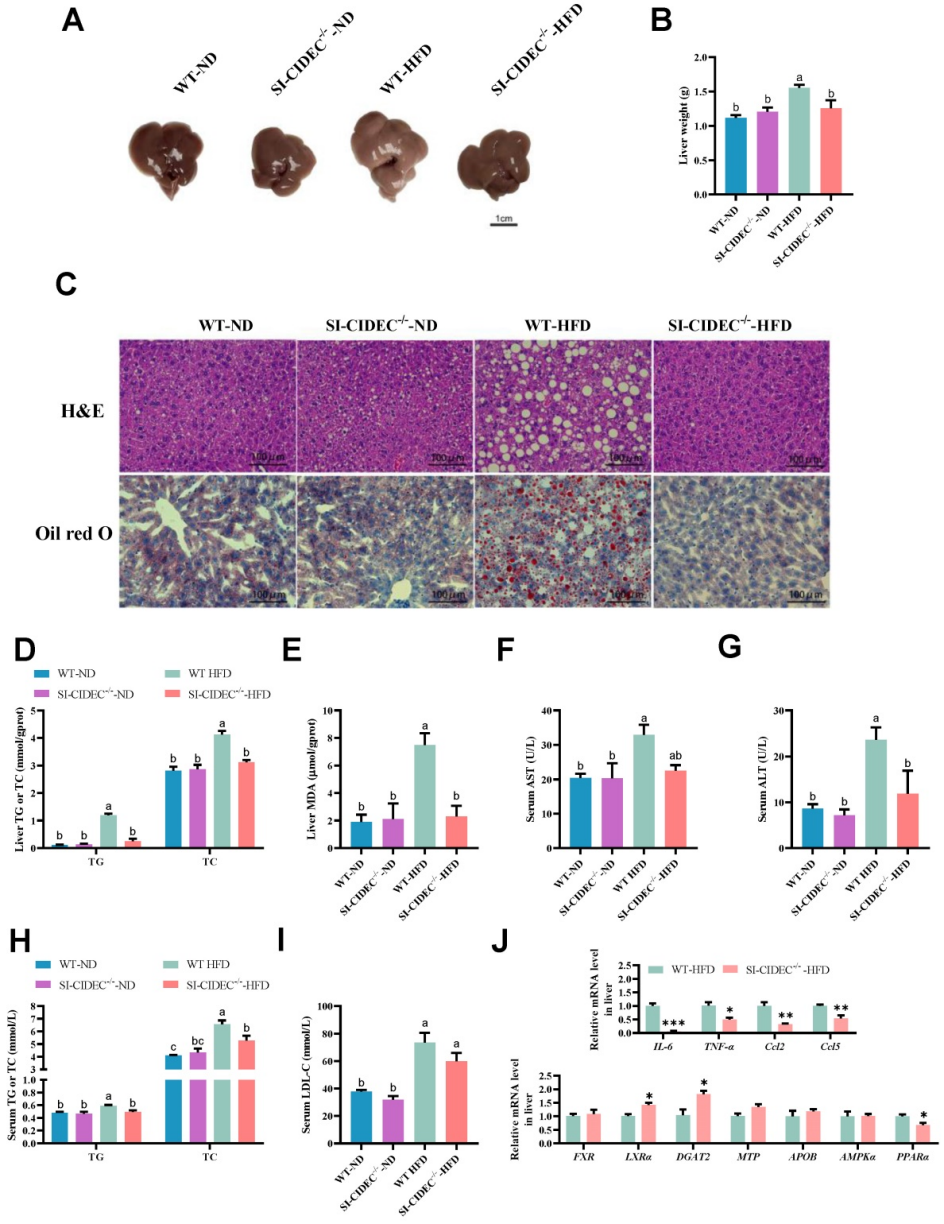
**SI-CIDEC^-/-^ improved diet-induced hepatic steatosis and hyperlipidemia risk. (A-I)** 22-week-old HFD/ND-fed mice (n=5-10) modeling from 8-week age was used. **(A)** Representative photographs of liver tissue; Scale bar: 1cm; **(B)** Liver weight;** (C)** Representative photographs of liver histology; Scale bar:100 µm (40× objective); **(D, E)** Liver TG (triglyceride), TC (total cholesterol) and MDA (malondialdehyde) concentrations; **(F-I)** Serum ALT (Alanine aminotransferase), AST (Aspartate aminotransferase), TG, TC and LDL-C (Low-density lipoprotein cholesterol) concentrations (n=4-10). Data are mean ± SEM. Mean values with different superscript letters differ significantly (*p* < 0.05).** (J)** mRNA expression of enzymes involved in liver lipid homeostasis and inflammation-related genes; SI-CIDEC^-/-^ and WT (n=3-4) on HFD were used; Data are normalized to *GAPDH* and mean ± SEM. Differences were considered significant at *p < 0.05.

As obesity often causes fatty liver, we investigated the effects of gut-specific knockout of CIDEC on HFD-induced fatty liver. Mice fed high-fat diets were larger and heavier than those fed an ND and liver tissue pathology was observed, WT-HFD mice had severe hepatic steatosis (Figure 3A-C), and the steatotic changes in SI-CIDEC**^-/-^**-HFD mice were not different from those in SI-CIDEC**^-/-^**-ND and WT-ND mice. Compared with WT-HFD mice, SI-CIDEC**^-/-^**-HFD mice had significantly reduced liver TG, TC, and MDA levels that were similar to the those in mice fed an ND (Figure 3D, E). In addition, SI-CIDEC**^-/-^**-HFD mice had significantly lower serum ALT than WT-HFD mice. Serum AST was also lower in SI-CIDEC**^-/-^**-HFD mice than in WT-HFD mice, but the difference was not significant (Figure 3G, H). The HFD increased serum TG, TC and LDL-C in WT mice (Figure 3H, I). Serum LDL-C was positively correlated with the risk of cardiovascular disease. As the SI-CIDEC**^-/-^**-HFD mice had lower TG, TC and LDL-C levels than the WT-HFD mice, small intestine-specific knockout of CIDEC may reduce cardiovascular disease risk by improving blood cholesterol composition. The mRNA expression of inflammation-related genes (IL-6, TNF-α, Ccl2, and Ccl5) was decreased, and the mRNA expression of lipid homeostasis-related genes (LXRα and DGAT2) was increased in the liver (Figure 3J), indicating that the secretion of very low-density lipoprotein was increased, and that the reason for the decrease of serum TG was decreased chylomicron (CM) secretion by the small intestine. The results indicate that SI-CIDEC**^-/-^** mice resisted diet-induced hepatic steatosis and changes in blood cholesterol.

### Energy metabolism in SI-CIDEC^-/-^ mice

**Figure 4:**
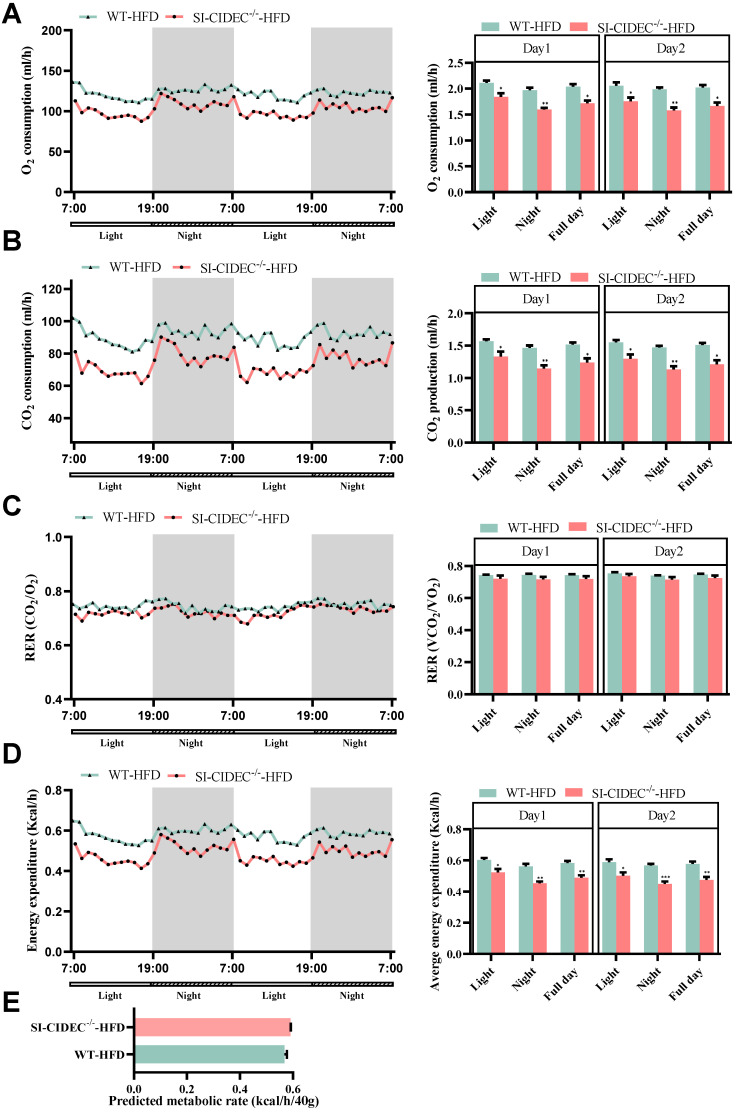
**Energy metabolism in SI-CIDEC^-/-^ mice. (A-D)** 17-week-old mice fed a high-fat diet from 8 weeks of age were used (both n = 4), data obtained during 2 days are shown. Each bar on the right was the mean during the light, night and whole day (light and night). **(A)** Whole body oxygen consumption (mL/h). **(B)** CO_2_ production (mL/h); **(C)** Respiratory exchange ratio (VCO_2_/VO_2_); **(D)** Energy expenditure (Kcal/h). Data are means ± SEM; **(E)** Predicted metabolic rate (Kcal/h/40 g), the method has been previously predescribed. (21) Data are means ± SEM; Differences were considered significant at *p < 0.05, **p < 0.01, and ***p < 0.001.

The energy expenditure in mice fed an HFD and housed in metabolic cages was measured as previously described 21. The night time O_2_ consumption, CO_2_ production, and energy metabolism of SI-CIDEC^-/-^ mice were significantly decreased compared with WT mice (Figure 4A, B, D), but differences in the respiratory exchange and predicted metabolic rates in the two genotypes were not significant (Figure 4C, E). However, CO_2_ production, O_2_ consumption, and energy consumption was decreased in SI-CIDEC^-**/**-^-ND mice compared with the WT-ND mice (Figure S1A-E). The results indicate that energy metabolism was not the reason for improved obesity and fatty liver in the small intestine-specific knockout mice.

### SI-CIDEC^-/-^ restricted intestinal absorption of lipids

**Figure 5:**
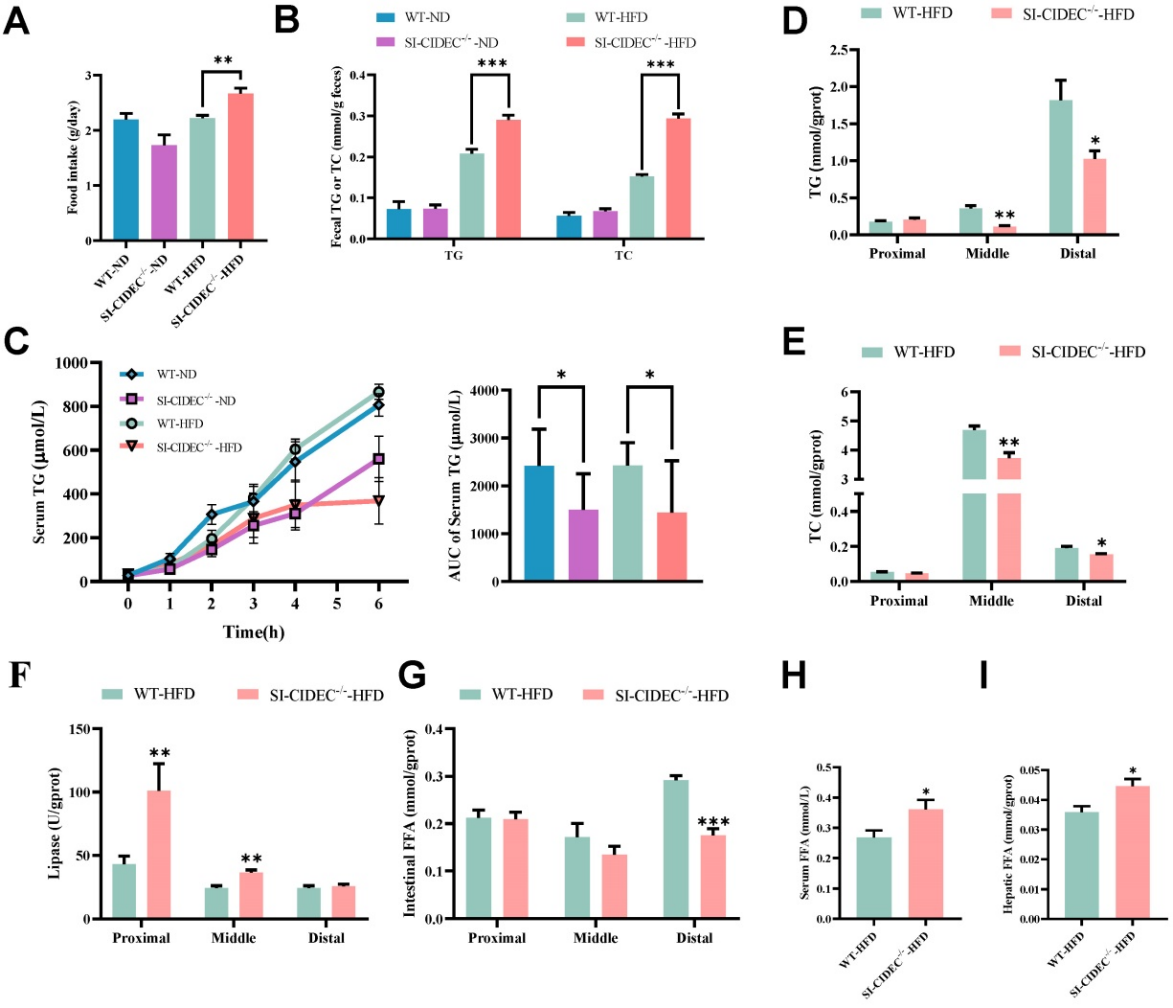
**SI-CIDEC^-/-^ restricts intestinal absorption of lipids. (A-I)** 22-week-old mice fed high-fat (HFD) or normal diets (n=5-10) beginning at 8 weeks of age.** (A)** Food intake of WT-ND (n=3), SI-CIDEC^-/-^-ND (n=3), WT-HFD (n=4) and SI-CIDEC^-/-^-HFD (n=3); **(B)** Fecal TG (triglyceride) and TC (total cholesterol) concentrations (n=4-5); **(C)** Postprandial serum TG concentrations in 0-6 h post-gavage with inhibition of lipase by tyloxapol (each group n=7); **(D-E)** Proximal, middle, and distal intestine in 22-week-old HFD fed mice (n=6-10) were used;** (D)** TG concentrations; Scale bar:100 μm (40× objective); **(E)** TC concentrations;** (F)** Lipase activity;** (G)** Intestinal free fatty acid (FFA) concentration; **(H)** Serum FFA concentration; **(I)** Hepatic FFA concentration; Data are means ± SEM. Differences were considered significant at *p < 0.05, **p < 0.01, ***p < 0.001.

To investigate whether the improvement of diet-induced obesity and fatty liver in SI-CIDEC^-/-^ mice was caused by altered intestinal digestion and absorption, we monitored food intake, fecal TG, and TC. They were significantly increased in SI-CIDEC^-/-^-HFD mice compared with WT-HFD mice (Figure 5A, B), and were not changed in ND mice. We modeled the postprandial state with an olive oil gavage after injection of tyloxapol, which showed that serum TG was significantly lower in SI-CIDEC^-/-^ mice than in WT mice, regardless of the diet (Figure 5C), consistent with reduced intestinal absorption in SI-CIDEC^-/-^. To determine how knockout of CIDEC impaired lipid absorption, we investigated changes in lipid accumulation, catabolism, and secretion. TG and TC in tissue sections from the middle and distal intestine were lower in SI-CIDEC^-/-^-HFD that in WT-HFD mice (Figure 5D, E and Figure S2A). Assays of lipase activity found increased lipolysis in the proximal and middle intestine of SI-CIDEC^-/-^ mice (Figure 5F), which may have been partly responsible for the reduced accumulation of intestinal lipids. In addition, lipase activity decreased from proximal to distal sections of the intestine of SI-CIDEC^-**/**-^-HFD mice and intestinal TG increased gradually from proximal to distal sections. Interestingly, changes in free fatty acid (FFA) levels in the intestine did not increase with increased lipase activity. Reduced FFAs were seen in the distal intestine (Figure 5G), and FFA levels in the circulating and liver were significantly elevated (Figure 5H, I).

We also examined changes in the mRNA expression of genes involved in lipid absorption (NPC1L1, SR-B1, and CD36), transport (FATP1 and FATP4), synthesis (DGAT2), secretion (APOB), catabolism (ATGL, HSL, and MGL) and metabolism (AMPKα and PPARα) in the intestine. Compared with WT-HFD mice, SI-CIDEC^-/-^-HFD mice had a significant increase in NPC1L1 transcripts and a decrease in monoglyceride lipase (MGL) transcripts in the proximal intestine (Figure S2B). increased SR-B1 and FATP1 and decreased MGL transcripts in the middle intestine (Figure S2C), and decreased SR-B1 and APOB in distal intestinal transcripts (Figure S2D). The data suggest that intestinal transport of cholesterol and FAs increased.

We tested the tight junction protein—ZO-1 protein expression in the intestine of two gene type mice under HFD. The results (Figure S3B) showed that ZO-1 protein expression was unchanged between two group. The permeability test also showed that overexpression of CIDEC-l did not affect the permeability of IPEC-J2 cells (Figure S3C). We conclude that there was no significant effect on intestinal permeability when changing CIDEC.

To detect the effect of intestinal CIDEC on gut microbiome, we examined changes of the microbiome in fresh feces from mice with 16S rRNA gene sequencing. No significant differences in alpha diversity were found following analysis with the ACE, Chao1, and Shannon indexes (Figure S4A-C). The two groups showed a separation along the PC2 axis of a principal coordinate analysis (PCoA) plot (Figure S4D). but no significant difference was obeserved in beta diversity of the two groups. Changes in the mean percentages of the intestinal microbiota at the phylum level or in the the *Firmicutes* to *Bacteroidetes* ratio were not significant (Figure S4E, F). Lefse analysis found significant differences in 15 species of bacteria in the two groups (Figure S4G, H), mainly belonging to the *Firmicutes*, *Bacteroidetes* and *Proteobacteria*. The results suggest that improvements in diet-induced obesity and hepatic steatosis did not result from changes of the gut microbiome.

### SI-CIDEC^-/-^ repressed lipid deposition by inhibiting AGPAT activity

**Figure 6:**
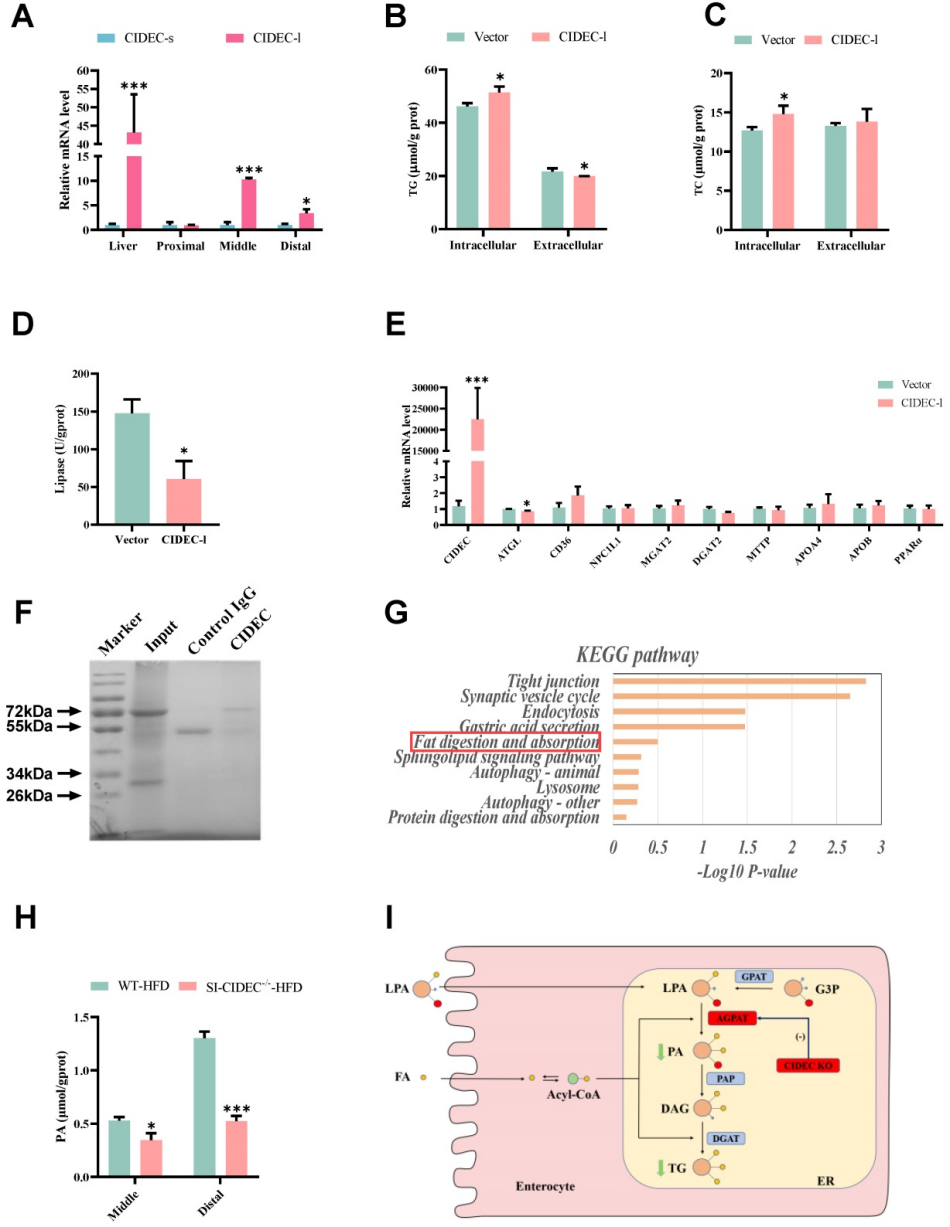
**CIDEC promotes lipid deposition by enhancing AGPAT enzyme activity. (A)** mRNA expression of two isoforms of CIDEC (n=3-4); 22-week-old mice modeled by feeding a high-fgat diet from 8 weeks of age were used. **(B-E)** After the introduction of CIDEC-l or vector, IPEC-J2 cells induced by high-fat for 48 hours were used (n=3-4);** (B)** Intracellular and extracellular triglyceride (TG) concentration; **(C)** Intracellular and extracellular total cholesterol (TC) concentration; **(D)** Lipase activity; **(E)** mRNA expression of enzymes involved in lipid absorption (*NPC1L1, SR-B1, CD36*), transportation (*FATP1, FATP4*), synthesis (*DGAT2*), secretion (*MTP, APOB*), lipolysis (*ATGL, HSL, MGL*) and oxidative metabolism (*AMPKα*, *PPARα*) in cells normalized to *GAPDH*; **(F)** SDS-PAGE stained by Coomassie brilliant blue. Protein immunoprecipitated from total intestinal protein of wild-type mice (no flox) fed a normal diet. Input, total intestinal protein; control IgG, immunoprecipitated protein using negative control mouse IgG antibody; CIDEC, immunoprecipitated protein using CIDEC antibody. **(G)** Intestinal CIDEC-interacting protein enrichment KEGG analysis; **(H)** Phosphatidic acid concentrations in the middle and proximal intestine of mice fed a high-fat diet (n=3-5). Data are means ± SEM. Differences were considered significant at *p < 0.05, ***p < 0.001. **(I)** Mechanism of CIDEC accelerating TG synthesis by the glycerol-3-phosphate pathway in the small intestine.

RT-PCR showed that long isoform, CIDEC-1, was the one that was primarily expressed in the intestine of WT-HFD mice (Figure 6A), which is similar to the findings in experimental pig models 18. In IPEC-J2 cells overexpressing CIDEC-l, the longer isoform of porcine CIDEC, treatment with oleic acid and palmitic acid (i.e. high-fat induction) resulted in IPEC-J2 with increased TG and TC and in lower extracellular TG than seen in cells transfected with empty vector (Figure 6B-C). Lipase activity was reduced (Figure 6D), and expression of *ATGL* mRNA was detected in IPEC-J2 cells transfected with CIDEC-l (Figure 6E).

To further investigate how CIDEC affected lipid absorption in the intestine, we isolated proteins that interact with CIDEC (Figure 6F), and identified them by coimmunoprecipitation and mass spectrometry (Co-IP/MS). KEGG database enrichment analysis identified one of the proteins (AGPAT) in the lipid digestion and absorption pathway (Figure 6G). Because the lipid levels changed in the mid and distal intestine of mice fed an HFD, we assayed phosphatidic acid concentrations in those two sections. The data indicated that the concentration of phosphatidic acid was significantly lower in the SI-CIDEC^-**/**-^-HFD mice than in WT-HFD mice (Figure 6H). AGPAT is abundant in the ER and catalyzes the conversion of lysophosphatidic acid to phosphatidic acid in the glycerol-3-phosphate pathway. After being dephosphorylated by PAP, phosphatidic acid is transformed into diacylglycerol (Figure 6I). As the glycerol-3-phosphate pathway accounts for 20-30% of TG synthesis during lipid absorption 22-25, which suggests SI-CIDEC^-**/**-^ decreased TG accumulation by inhibiting the catalytic activity of AGPAT.

## Discussion

Previous studies reported aggravated liver steatosis associated with increased serum TG in CIDEC-deficient, CIDEC^-**/**-/ob/ob^ double-deficient, CIDEC^-**/**-^/BATless, and adipose-specific knockout mice 12-17. The evidence suggests that CIDEC is required for adipose tissue absorption and storage of circulating lipids. CIDEC expression in visceral adipose tissue was significantly decreased in obese or obese-T2D subjects compared with nonobese subjects 26, 27. Here, we found that CIDEC protein expression in the intestine was reduced by the HFD (Figure S3A), analogous changes in CIDEC expression in the adipose tissue and the small intestine suggesting that CIDEC may play similar roles in those tissues. Our results showed that SI-CIDEC^-/-^ mice fed an HFD weighed less, had a smaller fat mass, much like previous studies, but the difference was that SI-CIDEC^-/-^ mice had normal livers without steatosis and without change in energy metabolism compared with WT mice fed an HFD. Intestinal CIDEC may thus be a suitable target for the treatment of obesity, with reduced lipid accumulation in critical metabolic tissues.

Impaired intestinal lipid absorption was shown by increased fecal TG concentration and decreased postprandial serum TG concentration in SI-CIDEC^-**/**-^ mice. In addition, no changes in liver, serum and fecal TG and TC concentrations of SI-CIDEC^-**/**-^-ND compared with WT-ND mice were seen. Reduced food intake and energy metabolism in SI-CIDEC^-**/**-^-ND mice were responsible for its decreased adipocyte size but unchanged adipose weight, fat mass, or body weight. Co-IP/MS revealed that AGPAT was a potential downstream target of CIDEC, CIDEC accelerated the synthesis of TG from G3P by enhancing the AGPAT activity.

As no change in the energy metabolism of SI-CIDEC^-**/**-^ mice fed an HFD was observed, impaired lipid absorption is most likely the main reason for improved obesity. What caused impaired lipid absorption in the small intestine of SI-CIDEC^-**/**-^ mice? Cholesterol, a precursor of bile acids, was reduced in SI-CIDEC^-**/**-^-HFD mice. Reduced bile acid secretion may be one of the reasons for the decreased lipid absorption that was observed. The digestion and absorption of lipids cannot be achieved without lipase, and inhibition of CIDEC has been reported to enhance *ATGL* activity, thus increasing lipolysis 3, 28, 29. The absence of intestinal *ATGL* was shown not affect lipid absorption and secretion in mice 3, but small intestine-specific overexpression of *ATGL* in mice has been shown to decrease intestinal TG without affecting the secretion of TG chylomicrons or changing body weight 30. The evidence shows that ATGL is not necessary for intestinal absorption in mice, and suggests that increased lipase activity in the intestine of SI-CIDEC^-**/**-^ mice was not directly responsible for the inhibition of intestinal absorption. As MGL-deficient mice have reduced feed conversion 31, 32, decreased MGL expression in the SI-CIDEC^-**/**-^ mouse intestine could affect lipid absorption. Lipid absorption is complicated and involves multiple pathways that could be influenced by intestinal CIDEC knockout.

Co-IP/MS identified AGPAT as a target protein of CIDEC in the regulation of intestinal lipid metabolism and homeostasis. Previous studies have shown that AGPAT1-deficient mice die before weaning and have reduced body weight, fat mass, and blood glucose 33. AGPAT2-deficient mice have congenital generalized lipodystrophy, develop severe fatty liver even if fed an ND, and do not form white and brown adipose tissue because of impaired adipose differentiation 34, 35. Downregulation of PPARγ and C/EBPβ transcription has been detected in AGPAT2-deficient mice, and CIDEC expression is known to be regulated by transcription factors including SREBP1c, PPARγ, C/EBP, and PPARα 36-39. Both AGPAT1 and AGPAT2 catalyze the synthesis of TG in the glycerol-3-phosphate pathway, and are expressed in the small intestine. In SI-CIDEC^-**/**-^ mice fed an HFD, the synthesis of phosphatidic acid, which is catalyzed by AGPAT, was significantly reduced, suggesting the interaction of CIDEC and AGPAT. The study found that CIDEC promoted lipid absorption in the mouse intestine that affected the homeostasis of systemic lipid metabolism. Small intestine-specific knockout of CIDEC improved but did not completely impair lipid absorption in mice by inhibiting AGPAT activity. Intestinal CIDEC may be a novel target for the treatment of obesity-associated diseases.

## Supplementary Material

Supplementary figures and table.

## Abbreviations

CIDEC: cell death-inducing DFFA-like effector C
Fsp27: fat specific protein
CIDEC-l: long isoform of CIDEC
SI-CIDEC^-/-^: small intestine-specific CIDEC
TG: triglycerides
WT: wild-type
AGPAT: 1-acylglycerol-3-phosphate-O-acyltransferase
MAG: 2-monoacylglycerol
DAG: diglyceride
FA: fatty acid
LPL: lipoprotein lipase
CE: cholesterol ester
ER: endoplasmic reticulum
MGAT: alpha-1,3-mannosyl-glycoprotein 2-beta-N-monoacetylglyceride acyltransferase
DGAT: diglyceride acyltransferase
TC: total cholesterol
LPA: lysophosphatidic acid
PA: phosphatidic acid
G3P: glycerol-3-phosphate
GPAT: glycerol-3-phosphate O-acyltransferase 1/2
PAP: phospholipid phosphatase 1

## References

[1] WHO. Prevalence of obesity among adults, BMI ≥ 30.

[2] Storch J, Zhou YX, Lagakos WS (2008). Metabolism of apical versus basolateral sn-2-monoacylglycerol and fatty acids in rodent small intestine. J Lipid Res.

[3] Korbelius M, Vujic N, Sachdev V (2019). ATGL/CGI-58-Dependent Hydrolysis of a Lipid Storage Pool in Murine Enterocytes. Cell Rep.

[4] Ho SY, Delgado L, Storch J (2002). Monoacylglycerol metabolism in human intestinal Caco-2 cells: evidence for metabolic compartmentation and hydrolysis. J Biol Chem.

[5] Phan CT, Tso P (2001). Intestinal lipid absorption and transport. Front Biosci.

[6] Ko CW, Qu J, Black DD (2020). Regulation of intestinal lipid metabolism: current concepts and relevance to disease. Nat Rev Gastroenterol Hepatol.

[7] Cifarelli V, Abumrad NA (2018). Intestinal CD36 and Other Key Proteins of Lipid Utilization: Role in Absorption and Gut Homeostasis. Compr Physiol.

[8] Xiao C, Stahel P, Carreiro AL (2018). Recent Advances in Triacylglycerol Mobilization by the Gut. Trends Endocrinol Metab.

[9] Williams PM, Chang DJ, Danesch U (1992). CCAAT/enhancer binding protein expression is rapidly extinguished in TA1 adipocyte cells treated with tumor necrosis factor. Mol Endocrinol.

[10] Puri V, Konda S, Ranjit S (2007). Fat-specific protein 27, a novel lipid droplet protein that enhances triglyceride storage. The Journal of Biological Chemistry.

[11] Nishino N, Tamori Y, Tateya S (2008). FSP27 contributes to efficient energy storage in murine white adipocytes by promoting the formation of unilocular lipid droplets. J Clin Invest.

[12] Toh SY, Gong J, Du G (2008). Up-regulation of mitochondrial activity and acquirement of brown adipose tissue-like property in the white adipose tissue of fsp27 deficient mice. PLoS One.

[13] Xu X, Park JG, So JS (2015). Transcriptional activation of Fsp27 by the liver-enriched transcription factor CREBH promotes lipid droplet growth and hepatic steatosis. Hepatology.

[14] Zhou L, Park SY, Xu L (2015). Insulin resistance and white adipose tissue inflammation are uncoupled in energetically challenged Fsp27-deficient mice. Nat Commun.

[15] Zhou L, Yu M, Arshad M (2018). Coordination Among Lipid Droplets, Peroxisomes, and Mitochondria Regulates Energy Expenditure Through the CIDE-ATGL-PPARα Pathway in Adipocytes. Diabetes.

[16] Tanaka N, Takahashi S, Matsubara T (2015). Adipocyte-specific disruption of fat-specific protein 27 causes hepatosteatosis and insulin resistance in high-fat diet-fed mice. J Biol Chem.

[17] Xu MJ, Cai Y, Wang H (2015). Fat-Specific Protein 27/CIDEC Promotes Development of Alcoholic Steatohepatitis in Mice and Humans. Gastroenterology.

[18] Li Y, Kang H, Chu Y (2018). Cidec differentially regulates lipid deposition and secretion through two tissue-specific isoforms. Gene.

[19] Zhang Z, Luo Z, Yu L (2022). Ruthenium 360 and mitoxantrone inhibit mitochondrial calcium uniporter channel to prevent liver steatosis induced by high-fat diet. Br J Pharmacol.

[20] Zhang Z, Pan T, Sun Y (2021). Dietary calcium supplementation promotes the accumulation of intramuscular fat. J Anim Sci Biotechnol.

[21] Müller TD, Klingenspor M, Tschöp MH (2021). Revisiting energy expenditure: how to correct mouse metabolic rate for body mass. Nat Metab.

[22] Ohlrogge J, Browse J (1995). Lipid biosynthesis. Plant Cell.

[23] Johnston JM, Rao GA, Lowe PA (1967). The separation of the alpha-glycerophosphate and monoglyceride pathways in the intestinal biosynthesis of triglycerides. Biochim Biophys Acta.

[24] Senior JR (1964). Intestinal Absorption of Fats. J Lipid Res.

[25] Johnston JM, Rao GA (1967). Intestinal absorption of fat. Protoplasma.

[26] Moreno-Navarrete JM, Ortega F, Serrano M (2014). CIDEC/FSP27 and PLIN1 gene expression run in parallel to mitochondrial genes in human adipose tissue, both increasing after weight loss. Int J Obes (Lond).

[27] Tan X, Cao Z, Li M (2016). TNF-alpha downregulates CIDEC via MEK/ERK pathway in human adipocytes. Obesity (Silver Spring).

[28] Singh M, Kaur R, Lee MJ (2014). Fat-specific protein 27 inhibits lipolysis by facilitating the inhibitory effect of transcription factor Egr1 on transcription of adipose triglyceride lipase. J Biol Chem.

[29] Grahn THM, Kaur R, Yin J (2014). Fat-specific protein 27 (FSP27) interacts with adipose triglyceride lipase (ATGL) to regulate lipolysis and insulin sensitivity in human adipocytes. J Biol Chem.

[30] Korbelius M, Vujic N, Kuentzel KB (2022). Enterocyte-specific ATGL overexpression affects intestinal and systemic cholesterol homeostasis. Biochim Biophys Acta Mol Cell Biol Lipids.

[31] Chon SH, Douglass JD, Zhou YX (2012). Over-expression of monoacylglycerol lipase (MGL) in small intestine alters endocannabinoid levels and whole body energy balance, resulting in obesity. PLoS One.

[32] Yoshida K, Kita Y, Tokuoka SM (2019). Monoacylglycerol lipase deficiency affects diet-induced obesity, fat absorption, and feeding behavior in CB cannabinoid receptor-deficient mice. FASEB J.

[33] Agarwal AK, Tunison K, Dalal JS (2017). Metabolic, Reproductive, and Neurologic Abnormalities in Agpat1-Null Mice. Endocrinology.

[34] Cortes VA, Curtis DE, Sukumaran S (2009). Molecular mechanisms of hepatic steatosis and insulin resistance in the AGPAT2-deficient mouse model of congenital generalized lipodystrophy. Cell Metab.

[35] Tapia PJ, Figueroa AM, Eisner V (2020). Absence of AGPAT2 impairs brown adipogenesis, increases IFN stimulated gene expression and alters mitochondrial morphology. Metabolism.

[36] Chen A, Chen X, Cheng S (2018). FTO promotes SREBP1c maturation and enhances CIDEC transcription during lipid accumulation in HepG2 cells. Biochim Biophys Acta Mol Cell Biol Lipids.

[37] Kim YJ, Cho SY, Yun CH (2008). Transcriptional activation of Cidec by PPARgamma2 in adipocyte. Biochem Biophys Res Commun.

[38] Danesch U, Hoeck W, Ringold GM (1992). Cloning and transcriptional regulation of a novel adipocyte-specific gene, FSP27. CAAT-enhancer-binding protein (C/EBP) and C/EBP-like proteins interact with sequences required for differentiation-dependent expression. J Biol Chem.

[39] Langhi C, Baldán Á (2015). CIDEC/FSP27 is regulated by peroxisome proliferator-activated receptor alpha and plays a critical role in fasting- and diet-induced hepatosteatosis. Hepatology.

[40] Jiang Q, Yin J, Chen J (2021). 4-Phenylbutyric acid accelerates rehabilitation of barrier function in IPEC-J2 cell monolayer model. Anim Nutr.

