# Supplementary material for: Small Intestine-specific Knockout of CIDEC Improves Obesity and Hepatic Steatosis by Inhibiting Synthesis of Phosphatidic Acid
Source: Int J Biol Sci. 2022 Sep 11;18(15):5740–52. doi: 10.7150/ijbs.74348 (PMC9576510; doi:10.7150/ijbs.74348)
Supplement: Supplementary file 1 — Supplementary figures and table. [file ijbsv18p5740s1.pdf]

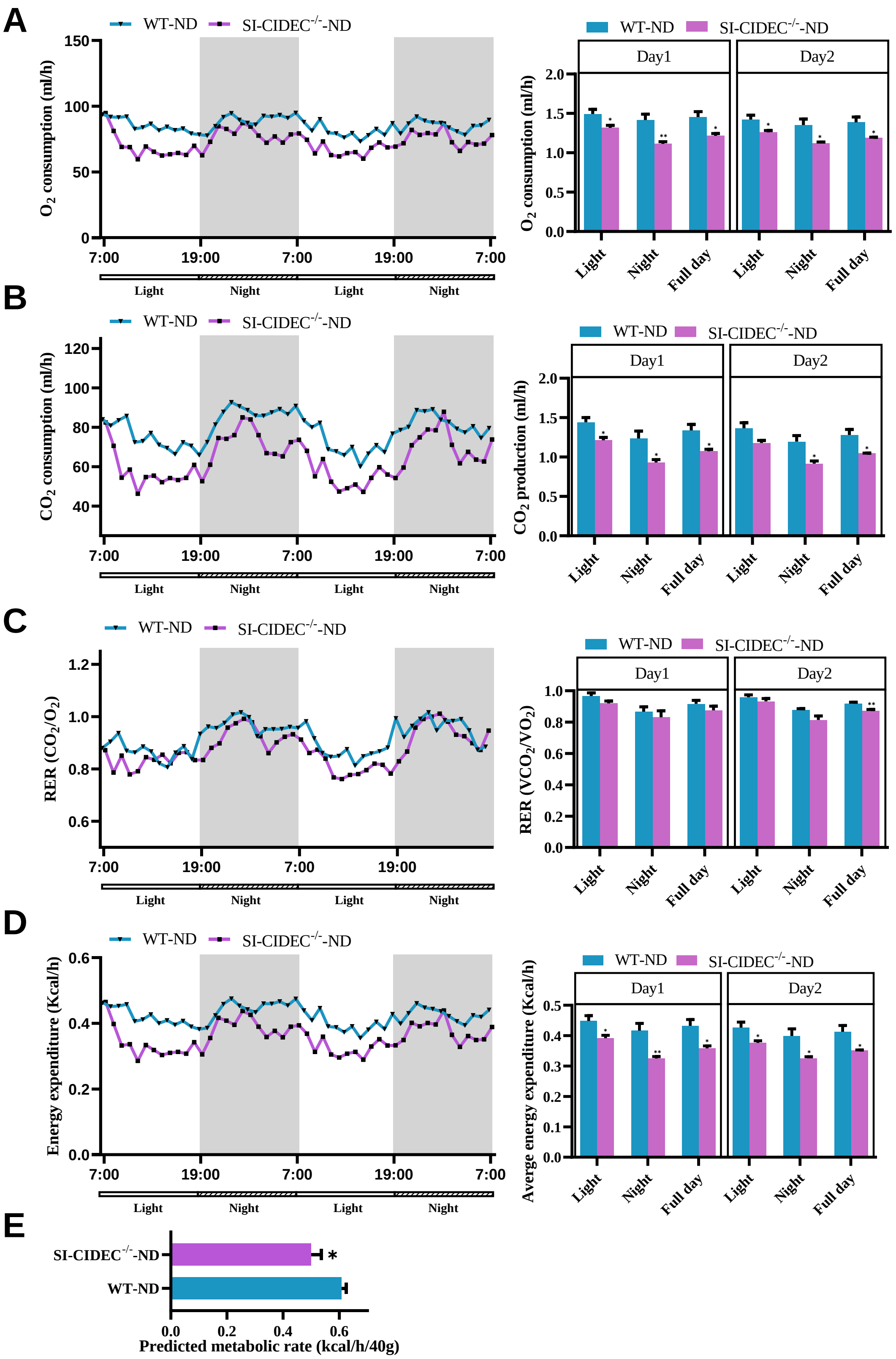

Figure S1. Energy metabolism in ND mice.

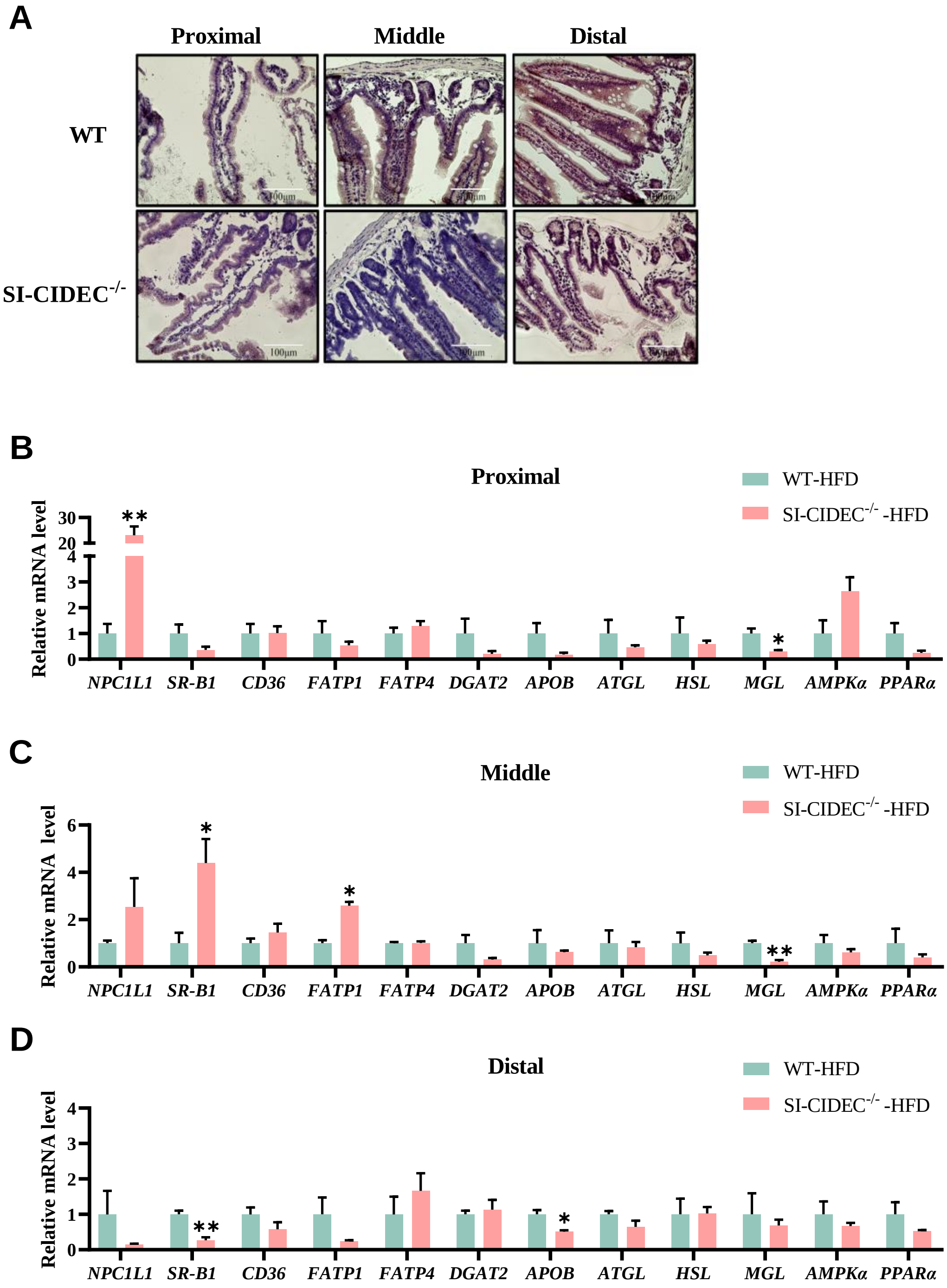

Figure S2. Intestinal histology and mRNA expression.

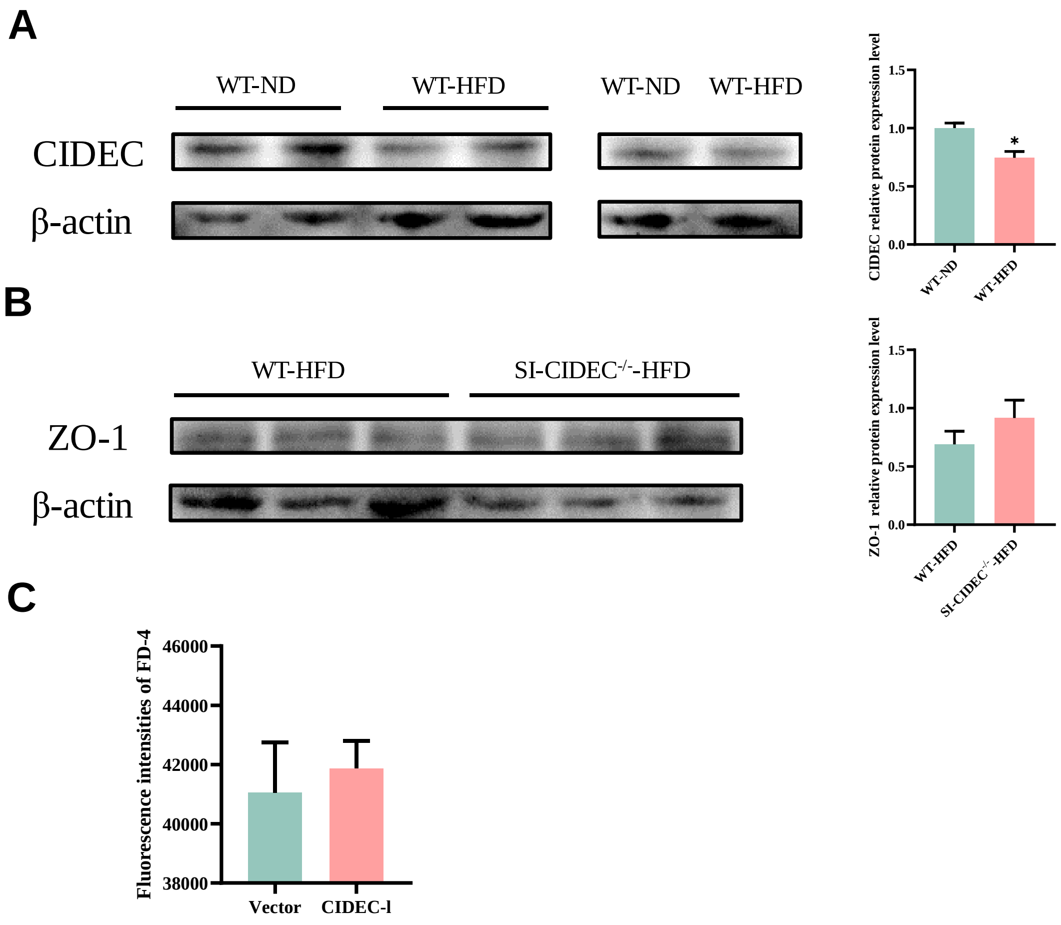

Figure S3. Effect of intestinal CIDEC on tight junctions.

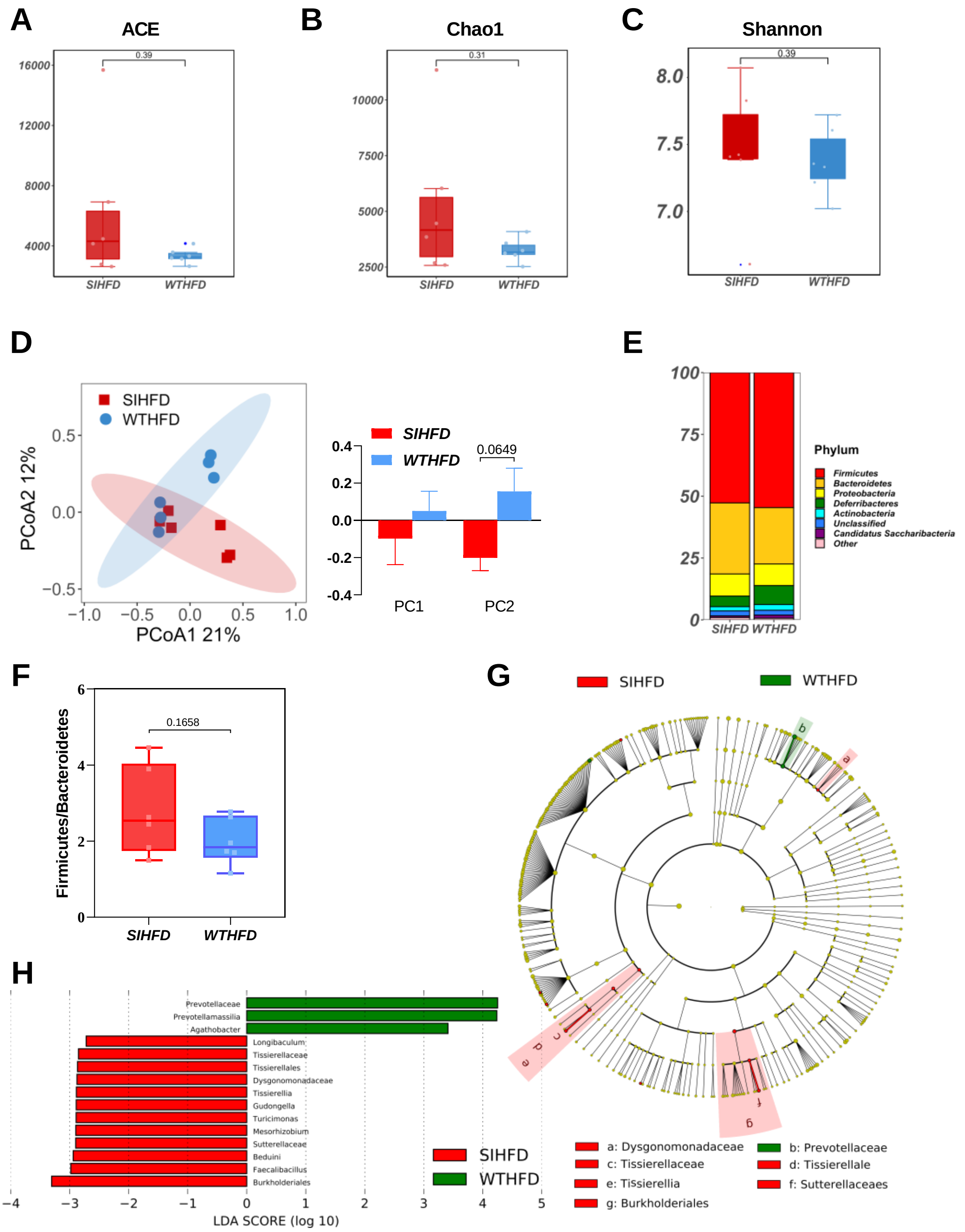

Figure S4. Gut microbiomes in mice of two genotypes fed a high-fat diet.

Figure S1. Energy metabolism in ND mice. (A–D) 21-week-old mice fed an ND from 8 weeks of age were used (both n = 4), data obtained during 2 days are shown. Each bar on the right was the mean during the light, night and whole day (light and night). **(A)** Whole-body oxygen consumption (mL/h). **(B)** CO_2 production (mL/h); **(C)** Respiratory exchange ratio (VCO_2/VO_2); **(D)** Energy expenditure (Kcal/h). Data are means ± SEM; **(E)** Predicted metabolic rate (kcal/h/40g) was determined as previously described (21). Data are means ± SEM; Differences were considered significant at *$p < 0.05$, **$p < 0.01$, *** $p < 0.001$.

Figure S2. Intestinal histology and mRNA expression. (A–D) 22-week-old mice fed high-fat (HFD) or normal (ND) diets (n=5–10) modeling from 8 weeks of age were used. **(A)** Representative photographs of histology **(**stained by Oil red O**)**; **(B–D)** mRNA expression of enzymes involved in lipid absorption (*NPC1L1, SR-B1, CD36*), transportation (*FATP1, FATP4*), synthesis (*DGAT2*), secretion (*APOB*), lipolysis (*ATGL, HSL, MGL*), and oxidative metabolism (*AMPKα, PPARα*) in the **(B)** proximal, **(C)** middle and **(D)** distal intestine; SI-CIDEC$^{-/-}$ and WT (n=3–4) on HFD were used; Data are normalized to *GAPDH*; Data are means ± SEM. Differences were considered significant at *$p < 0.05$, **$p < 0.01$, ***$p < 0.001$.

Figure S3. Effect of intestinal CIDEC on tight junctions. (A) CIDEC protein expression in the intestine of WT mice fed different diets. Intestinal tissues were obtained from 22-week-old mice fed normal or high-fat diets (n=3); **(B)** ZO-1 tight junction protein 1 (TJP1) protein expression in the intestine of two genotype mice fed an HFD. Intestinal tissues were obtained from 22-week-old mice fed an ND or an HFD (n=3); **(C)** Permeability assay in IPEC-J2 cells. FD-4 (FITC-Dextran 4kDa) fluorescence intensities of the culture medium in the basal compartment are shown. The methods have been previously described 40; Data are means ± SEM. Differences were considered significant at *$p < 0.05$, **$p < 0.01$, *** $p < 0.001$.

Figure S4. Gut microbiomes in mice of two genotypes fed a high-fat diet. (A–H) Fresh feces from 22-week-old SI-CIDEC$^{-/-}$ mice and WT mice fed HFDs were used for 16S rRNA sequencing, n=6, SI-CIDEC$^{-/-}$(SIHFD), WT-HFD (WTHFD) **(A-C)** alpha diversity of the gut microbiota in the two groups determined by the ACE, Chao1, and Shannon indices; **(D)** beta diversity of the gut microbiota between two groups indicated by principle coordinate analysis (PCoA). The PCoA plot was generated by OTU metrics based on the Bray-Curtis similarity. The values of PC1 and PC2 are shown in bar plots and expressed as means ± SEM, significant differences were detected by Mann-Whitney U-tests; **(E)** Mean percentage of the total population at the phylum level; **(F)** *Firmicutes* to *Bacteroidetes* ratio; data were expressed as means ± SEM, significant differences were detected by two-tail *t*-tests. **(G)** Taxonomic cladogram generated from LEfSe of 16S rRNA sequencing data. Enriched taxa of WT-HFD (green) and SI-CIDEC$^{-/-}$-HFD (red) groups were shown. Size of each circle is proportional to the taxon's abundance. **(H)** Linear discriminant analysis (LDA) effect size method was performed to compare taxa between two groups. The bar chart listed the significantly differential taxa based on effect size (log_{10} LDA score > 2). Enriched taxa in

SI-CIDEC$^{-/-}$-HFD (negative LDA score), and enriched taxa in WT-HFD (positive LDA score).

Table S1. RT-PCR Primer sets

Gene Name	Primer Sequence (5´-3´)
CIDEC (mus)	F-AGCCCTCCTCCCTCCTC<br>R-TCCTTGGTGCTGTGCTGT
ATGL (mus)	F-GTCCTTCACCATCCGCTT<br>R-CTCTTGGCCCTCATCACC
HSL (mus)	F-TTCAGACAGCCCCGAGA<br>R-TGACATCAGAGGGTGTGGA
MGL (mus)	F-GGCTGGACATGCTGGTATT<br>R-TCGGGGTAGTCCTTCTGG
GAPDH (mus)	F-TGTTTCCTCGTCCCGTAGA<br>R-ATCTCCACTTTGCCACTGC
NPC1L1 (mus)	F-AGATGGAGCCGAGTTGC<br>R-CCAGAGAGGAGGGGACA
SR-B1 (mus)	F-TGCTTTTATGAACCGCACA<br>R-CCCAACAAACAGGCCAA
CD36 (mus)	F-TGGTGCTGTCATTGGAGCAGT<br>R-TGTCTGTAAACTTCCGTGCCTGT
FATP4 (mus)	F-TTCATCAAGACGGTCAGGCG<br>R-AGACGGTGGCAGCGAATAAG
MTP (mus)	F-AAATCGGGTAACCGTGGTAATAA<br>R-AGGCAAACTAAGAATGGGTACTGA
APOB (mus)	F-CGGATTCAAGAAGCTCCACC<br>R-GGACATGCGGCAGCAAACT
DGAT2 (mus)	F-ACCCGACCCAGAAAGACA<br>R-TTCACCTCCAGCACCTCA
AMPKα (mus)	F- GGACTTACTTGTTGGATTTCCG<br>R- CCTTTGGCAAGATCGATGTTG
PPARα (mus)	F- ATGCCAGTACTGCCGTTTTC<br>R- ACACGACCTGAAAGATTCGG

IL-6(mus)	F- ACAGAAGGAGTGGCTAAGGA<br>R- AGGCATAACGCACTAGGTTT
TNF-α(mus)	F- CGCTGAGGTCAATCTGC<br>R- GGCTGGGTAGAGAATGGA
Nos2(mus)	F-GTGGTGACAAGCACATTTGG<br>R-AAGGCCAAACACAGCATACC
Ccl2(mus)	F-GGGATCATCTTGCTGGTGAA<br>R-AGGTCCCTGTCATGCTTCTG
Ccl5(mus)	F-TGCTTTGCCTACCTCTCC<br>R-CACACACTTGGCGGTTC
Adipoq(mus)	F-TGCTTTGGTCCCTCCAC<br>R-AGTGCCATCTCTGCCATC
CIDEC (sus)	F- GTGGCCCGTGTAACCTTC<br>R- AAAGCAGCGCAGATCGTAG
GAPDH (sus)	F- CTGTTCGGTTGTGGATCTGA<br>R-TGACGAAGTGGTCGTTGAGG
ATGL (sus)	F- CTGACGGAGCAGGTGGAG<br>R- CCCAGCGAGAGGCTGTT
CD36 (sus)	F- CCTTCACTGTTCTCAATCTGG<br>R- TGTGGTAGGAATAGGGTATGG
NPC1L1 (sus)	F- CAGCGGGATTCTGTCCT<br>R- CTGGTGTTGTGTGGGTTG
MGAT2 (sus)	F- AAGCGGAAGGTGCTGAT<br>R- CTCCAGAGGACGAAGCC
DGAT2 (sus)	F- TGCTGCGGGAGTACCTG<br>R- TGCTGCGGGAGTACCTG
MTTP (sus)	F-AAATCGGGTAACCGTGGTAATAA<br>RAGGCAAACTAAGAATGGGTACTGA
APOB (sus)	F-CGGATTCAAGAAGCTCCACC<br>R-GGACATGCGGCAGCAAACT

PPARα (sus)	F- GTTCGCCAAGTCCATCC<br>R- GCATCCCGTCCTTGTTC
